# Focused ultrasound and Alzheimer’s disease A systematic review

**DOI:** 10.1590/1980-57642018dn12-040003

**Published:** 2018

**Authors:** Rodrigo Marmo da Costa e Souza, Inaê Carolline Silveira da Silva, Anna Beatriz Temoteo Delgado, Pedro Hugo Vieira da Silva, Victor Ribeiro Xavier Costa

**Affiliations:** 1 Neurosurgeon. Departamento de Psicologia, Programa de Neurociências Cognitiva e do Comportamento, Universidade Federal da Paraíba, João Pessoa, Paraíba, Brazil; 2 Medical student. Departamento de Medicina, Universidade Federal da Paraíba, João Pessoa, PB, Brazil; 3 Medical student. Departamento de Medicina, Faculdade de Ciências Médicas da Paraíba, Cabedelo, PB, Brazil; 4 Departamento de Medicina, Liga de Neurologia e Neurocirurgia Funcional da Paraíba, Cabedelo, PB, Brazil.

**Keywords:** ultrasonic therapy, Alzheimer´s disease, amyloid-beta peptides, terapia por ultrassom, doença de Alzheimer, peptídeos beta-amiloides

## Abstract

Alzheimer’s disease (AD) affects millions of people, however, there is still no effective treatment. The use of focused ultrasound with microbubbles (FUS-MB) for the opening of the blood-brain barrier has been recently studied and may become a promising therapeutic target.

**Objective:**

To discuss the use of FUS-MB for the treatment of AD and to present some of the techniques used.

**Methods:**

A systematic review was performed of MEDLINE/PubMed and Biblioteca Virtual em Saúde (BVS) services, using the keywords: focused ultrasound, Alzheimer, amyloid-b. Original articles were included in the study; studies that did not focus on Alzheimer’s treatment were excluded.

**Results:**

Fifteen original studies were selected. Preclinical trials were able to reduce amyloid-b plaques and tau phosphorylation, improving cognitive performance in AD animals.

**Conclusion:**

The results are very promising, but the therapy still requires maturation. Further studies are needed to systematize all the techniques used and their effects in order to enable use in humans.

## RESUMO

A doença de Alzheimer (DA) afeta milhões de pessoas, porém, ainda não há um tratamento efetivo. O uso de ultrassonografia focalizada com microbolhas (FUS-MB) para a abertura da barreira hematoencefálica (BHE) tem sido objeto de estudos recentes, podendo tornar-se um alvo terapêutico bastante promissor.

**Objetivo:**

Discutir o uso da FUS-MB para o tratamento da DA e apresentar algumas das técnicas utilizadas.

**Métodos:**

Revisão sistemática através dos serviços MEDLINE/PubMed e Biblioteca Virtual em Saúde (BVS), através das seguintes palavras-chaves: focused ultrasound, Alzheimer, amyloid-b. Foram incluídos no estudo artigos originais. Foram excluídos estudos que não tinham o tratamento do Alzheimer como foco.

**Resultados:**

Foram selecionados 15 estudos originais. Os resultados pré-clínicos são positivos para redução de placas amiloides, fosforilação da proteína tau e sobre desempenho cognitivo de animais com DA.

**Conclusão:**

Os resultados são promissores, mas terapia ainda necessita de aperfeiçoamento, necessitando de mais estudos que sistematize todas as técnicas e seus efeitos, para ser possível o seu uso em humanos.

Alzheimer’s disease (AD) is the leading cause of dementia in adulthood and is defined as a progressive multifactorial neurodegenerative disorder. Histologically, it is characterized by intracellular neurofibrillary tangles and deposits of extracellular amyloid protein that contribute to the formation of senile plaques.^1^ It is estimated that about 10% of people over 70 years old have significant memory loss and more than half of these individuals have AD.^2^ The prevalence increases with aging, which characterizes age as the greatest risk factor for the disease. Between 60 and 64 years old, the prevalence is 0.7%, and after 65 years old the prevalence doubles every five years, reaching 38.6% in nonagenarians.^3^ Currently, AD is a non-curable disease. The available drugs, acetylcholinesterase inhibitors (rivastigmine, galantamine, donepezil) and N-methyl-d-aspartate receptor antagonist (memantine) have minimal impact on the disease, serving to slow progression, providing only symptomatic relief. The absence of effective treatment is probably due to a lack of understanding of AD’s complex pathogenic mechanism.^1^ Unfortunately, most of the Phase 2 and 3 trials using immunotherapies, “anti-aggregants”, or related agents have been unsuccessful. Important progress has been made regarding AD’s pathophysiology, with new therapeutic targets emerging.

The opening of the blood-brain barrier by focused ultrasound (FUS) with contrast microbubbles (MBs) has been studied in recent years as a therapeutic option for AD.^4^ Ultrasonography is the most widely used diagnostic imaging method in the clinical field, mainly for the abdomen, female genitals and Doppler vascularization.^5^ In 1955, it was predicted that focused ultrasound would have a major impact on neurosurgical treatment, as it was used with a high intensity for irreversible tissue ablation in areas located in the brain for the treatment of chronic pain, Parkinson’s disease and movement disorders. However, it was noted that low-intensity pulsed focused ultrasound (LIFUP) caused less damage; also, the possibility of neuronal excitation and inhibition was perceived.^6^

Ultrasound can be used with contrast microbubbles, considering that they have been shown highly beneficial for evaluating tumors and microcirculations, for identifying some types of lesions and also to evaluate the therapeutic response.^5^ The act of applying sound (or ultrasound), to agitate particles is known as sonication. Regarding use in the neurological clinic, when microbubbles were introduced before the sonication, it was noted that there was no acute neuronal damage in the open ultrasonographic focus, limiting the effects to vascularization while protecting the exposed area. This protection can be potentialized in LIFUP, because the energy and pressure is lower than those used for ablative purposes and also owing to the phenomenon of localized cavitation generated when the microbubbles are injected, leading to a mechanism of stretching endothelial cell membranes, resulting in transient opening of the blood-brain barrier for a few seconds.^4^^,^ 7 The blood-brain barrier prevents potential transport of immunobiological agents or other types of drugs; therefore, studies have been done on how to increase its permeability temporarily for the administration of immunotherapeutic drugs aimed at the clearance of beta-amyloid plaques, which are present in neurodegenerative disorders, such as Alzheimer’s disease. FUS with contrast MBs has been reported in several studies involving rats, rabbits and monkeys for this purpose.^4^

The aim of this study was, through a systematic review, to explore the main results of all discussions and present a synthesis of the pre-clinical studies conducted to date, presenting some of the techniques used and discussing the effectiveness of the use of FUS-MB for the treatment of Alzheimer’s disease.

## METHODS

This study is a systematic review. Electronic searches were conducted on the Medical Literature Analysis and Retrieval System Online (MEDLINE) and Literatura Latino-Americana em Ciências da Saúde (LILACS) databases, through PubMed and Biblioteca Virtual de Saúde (BLS). The following keywords were used, in English and Portuguese: “Focused ultrasound” AND “Alzheimer”; “Focused ultrasound” AND “Amyloid-b”, in both search services. No studies were found in the Portuguese language. This review includes only original studies. The exclusion criteria were: review articles, editorials, newspaper articles, expert opinion or commentary and studies that did not have Alzheimer’s treatment as their main focus. The last search was carried out on September 20^th^ of 2018. Two reviewers independently assessed each study against predefined eligibility criteria. A third reviewer resolved any disagreement. After application of the criteria, 15 studies were selected, read in full and analyzed regarding the objectives, type of study, animal models used and key results.

Although the quality of the methods was the core of this review, there is always a small risk of selection bias. Having at least two assessors to select relevant reports and extract data reduces the potential selection and extraction bias and also decreases the possibility of accidental exclusion of relevant reports and inaccurate extraction of irrelevant data which may lead to distorted conclusions.

## RESULTS

**Figure 1:**
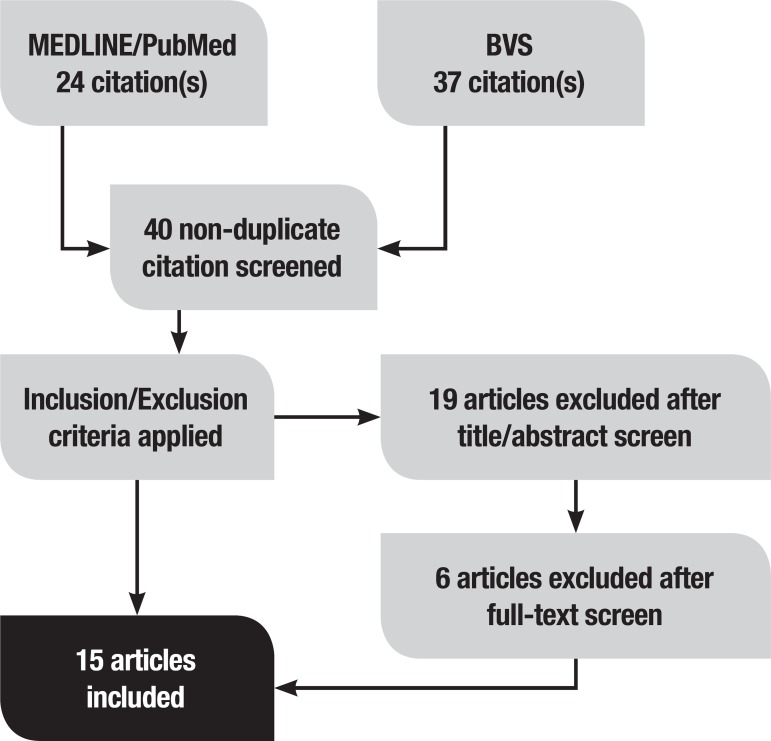
Selection of the studies evaluated

The search retrieved 61 articles, 24 on PubMed and 37 on BVS. Of these 61 studies, 40 non-duplicate articles were found. After applying the inclusion and exclusion criteria mentioned above, 19 articles were excluded based on title and abstract screen; a further 6 articles were excluded after full-text screening. As shown in Figure 1, a total of 15 studies were selected. The selected articles are summarized in Table 1 under the following criteria: authors, year of publication, animal model used and significant results.

**Table 1:** The use of focused ultrasound (FUS) to open the blood-brain barrier (BBB) in the treatment of Alzheimer's disease (AD): a list of pre-clinical studies analyzed and their results.

Author, year	Animal model	Results
Raymond et al., 2008^8^	Mouse	Sonication, with temporary interruption of the BBB, allowed the entry of therapeutic agents and molecular imaging agents.
Choi et al., 2008^9^	Mouse	FUS allowed the opening of BBB and the entry of molecules by microbubbles. The extent of the opening and closing time of the BBB depend on the region of the brain.
Jordão et al., 2010^10^	Mouse	FUS associated with administration of anti-b-amyloid antibody (BAM 10) in transgenic mice (TgCRND8) promoted a significant reduction of b-amyloid peptide plaques.
Konofagou et al., 2012^11^	Mouse	FUS with microbubbles targeting the hippocampus demonstrated increased BBB permeability by at least two orders of magnitude. Therapeutic molecules that improve cognition and brain aging successfully crossed the BBB. BBB permeability depended on the pressure used and the size of the microbubbles.
Jordão et al., 2013^12^	Mouse	After a single FUS session, b-amyloid plaque size was reduced by 20%, while the total plaque surface was reduced by 13%. Histological studies showed the entry of endogenous antibodies into the b-amyloid plaque and the activation of microglia and astrocytes.
Burgess et al., 2014^13^	Mouse	After the use of FUS, the permeability of the vessels of the transgenic mice (TgCRND8) and non-transgenic were compared. Transgenic vessels with amyloid plaques proved much more impermeable and had a smaller diameter after FUS.
Burgess et al., 2014^14^	Mouse	After sonication, there was a 99% improvement in the spatial memory activity of transgenic mice (TgCRND8), as well as a 250% increase in the number of new neurons in the hippocampus with longer and arborized dendrites.
Nisbet et al., 2017^15^	Mouse	Administration of 2N tau isoform-specific single chain antibody fragment (RN2N), combined with the use of FUS in scanner mode, significantly improved brain distribution and efficacy.
Alecou, Giannakou & Damianou 2017^16^	Rabbit	Administration of antibodies to b-amyloid protein reduced the number of plaques from 200 to 170/cm^2^, while the concomitant use of FUS and antibodies decreased levels from 200 to 78/cm^2^. Repeated application of FUS increased the reduction of b-amyloid plaques.
O'Reilly et al., 2017^17^	Rat	A study was performed to analyze the relationship between the opening volume of BBB and its closing time. Closing time of the BBB was independent of opening volume. It was suggested that larger volume opening can be done safely.
O'Reilly et al., 2017^18^	Dog	The opening of the BBB from a complete hemisphere of the brain by sonication was well tolerated by beagles (9-11 years old), indicating the clinical safety of the method. There was no significant reduction in b-amyloid load. Further studies are needed to determine whether there are benefits in natural pathologies associated with b-amyloid plaques.
Liu et al., 2018^19^	Mouse	This study aimed to analyze the association between FUS with anti-b-amyloid antibody (BAM-10) and scyllo-inositol. There was no summation of the benefits of FUS with BAM-10 associated with scyllo-inositol. Both were efficient alone or combined, reducing b-amyloid plaque and increasing astrocyte activation, but were not more effective when used together.
Hsu et al, 2018^20^	Mouse	This study aimed to evaluate whether the use of FUS-induced BBB opening could enhance the delivery of GSK-3 inhibitor, which would promote an additive effect on b-amyloid clearance, as well as reduce its synthesis in transgenic AD mice models. The procedure was performed unilaterally, using the contralateral hemisphere as a reference. Immunohistochemistry showed that GSK-3 inhibitors reduced GSK-3 activity by up to 61.3% with the addition of FUS, and autoradiography showed significant b-amyloid reduction.
Eguchi et al, 2018^21^	Mouse	Therapeutic FUS in the hippocampus exerts a neuroprotective action on dementia. The study evaluated the efficacy and safety of LIPUS throughout the brain in mice with 2 types of dementia: vascular dementia and Alzheimer's dementia. In both models, LIPUS therapy substantially improved cognitive deficits (labyrinth Y test and/or passive evasion test), improved cerebral blood flow and regulated endothelial related genes.
Xu et al, 2018^22^	Mouse	The failure of many clinical trials suggests inefficacy in the treatment of AD using only one target. This study used multiple targets through a nano-drug protoporphyrin IX (PX) modified oxidized mesoporous carbon (OMCN) nanospheres (PX @ OMCN @ PEG (OP) @RVGs), which appears to be an efficient inhibitor of phosphorylated tau. Also, PX, together with FUS, significantly reduced b-amyloid plaque aggregation.

FUS: Focused Ultrasound; BBB: Blood-Brain Barrier; GSK-3:Glycogen synthase kinase 3.

The use of FUS to treat AD is a recent technique. The first preclinical trial was published less than a decade ago (2008) and 53.33% of the articles selected were submitted between 2017 and 2018. All of the original studies so far are in the preclinical animal model phase.

**Figure 2:**
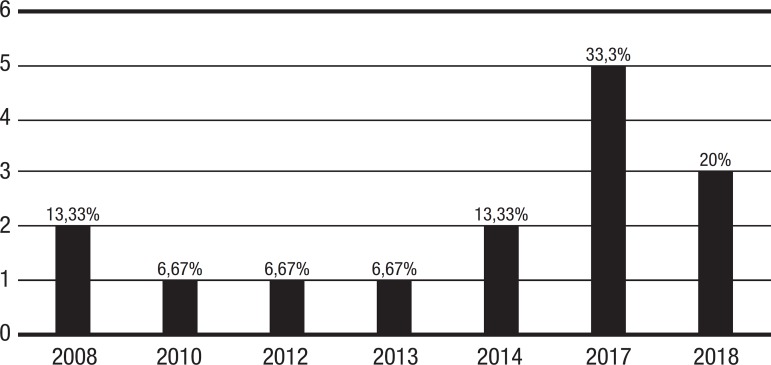
Number of pre-clinical studies published by year

Figure 2 shows that interest in this subject has risen over the last few years. Considering the articles selected, 2017 was the year with the largest number of publications, totaling 5 (33.3%) studies; 2018 was the second, with 3 (20%) publications up until the present date. 2008 and 2014 had 2 (13.3%) publications each, while 2010, 2012 and 2014 had only 1 (6.67%) article published each.

One of the difficulties in correlating the studies was the distinct aspects regarding the treatment of Alzheimer’s disease. Some authors sought to remove b-amyloid plaques, while others were only aiming to increase the permeability of BBB to allow the entry of drugs and substances to treat the disease. Different therapeutic targets, frequencies and microbubble sizes were used, hampering meaningful comparison of the studies.

## DISCUSSION

The blood-brain barrier (BBB) is characterized by a highly specialized composition of endothelial and perivascular structures, which differs from the barrier between the peripheral vasculature and other organs in the body mainly due to its tight junctions between adjacent endothelial cells.^23^ The endothelial cells are connected to each other by transmembrane proteins, creating tight junctions, and are supported by a basal lamina and a complex cellular system of neurons, astrocytes, microglia and pericytes, which function together as the BBB.^24^

This composition limits the transport of substances to the brain, precluding free exchanges of solutes between blood and brain.^24^ An exception to this rule are small lipid-soluble molecules with less than 400 Daltons (6.64 × 10^–19^ milligrams) in weight, which can cross the BBB unassisted, via lipid-mediated diffusion.^11^^,^ 24 This organization makes it difficult for most therapeutic agents to reach the brain tissue, therefore conferring a restriction on treatment approaches for neurodegenerative diseases, such as Alzheimer’s disease.^11^

Numerous methods have been developed to circumvent the BBB, such as novel drugs for improved access through the BBB, surgical intervention for delivery of drugs to the brain and the use of chemical agents or other techniques to increase the BBB permeability.^23^

The use of focused ultrasound (FUS) associated with microbubbles is one of the techniques that has been developed to temporarily and reversibly increase the permeability of the BBB,^8^^,^ 17 ^,^ 18 with the aim of improving drug delivery to the brain.

FUS localizes ultrasound energy to a millimeter-sized focal region, defined as the sonicated area (area of highest energy), which is affected by the ultrasound beam while the surrounding regions remain relatively unaffected.^9^ The method works by converting electrical energy into mechanical motion by the piezoelectric material of the transducer, thus generating ultrasound, which propagates through the skull and brain.^23^

Microbubbles (MB) are injected intravenously at the onset of sonication^23^ or 10 seconds prior to the start of each sonication.^25^ When the intravascular MB enter the ultrasound field, they expand and contract at the frequency of the ultrasound wave.

This leads to interaction with the endothelial cells and subsequent BBB disruption. The oscillating microbubbles are thought to stretch the blood vessel walls to induce BBB disruption; however, the precise physical mechanisms are unclear.^23^ Studies have postulated that oscillating microbubbles can be thought of as acoustic focusing agents that produce microstreaming, which causes vessel wall displacement.^26^

At high enough stresses, endothelial cells eventually detach or lose membrane integrity.^27^ However, intravenous administration of microbubbles allows the BBB to be opened using a significantly reduced acoustic power, over 100 times less than that required to produce thermal damage in the tissue.^23^ This is possible because the opening is not related to the intensity of the bubble collapse, but to oscillations of the MB with the endothelium, leading to the opening of the tight junctions and initiation of transcellular transport.^28^

The use of focused ultrasound associated with microbubbles (FUS-MB) has many advantages as a method to circumvent the BBB, such as being noninvasive, targeted, transient and safe.^23^ Monitoring of the BBB opening procedure may be done by simple magnetic resonance imaging (MRI) or by MRI contrast agents.^11^ This association is known as MRI-guided focused ultrasound (MRgFUS).

In addition, despite the previous knowledge that FUS-MB allows focusing of transport in precise anatomical regions, a study in Massachusetts that used MRgFUS-MB focusing on the hippocampus, also evidenced the inadvertent passage of drugs into the thalamus and cortex. As a possible solution to these complications, the authors suggested the use of a larger diameter transducer with a tighter focal volume.^8^

There have been many studies associating the use of MRgFUS plus MB with the possibility of treating Alzheimer’s disease (AD). The current accepted pathophysiology of AD is based on the amyloid-b (Ab) cascade hypothesis: Ab oligomer aggregates, formed by an imbalance between production and clearance of Ab peptide, are considered responsible for the neuronal and vascular degeneration. Ab oligomers also induce oxidative damage and promote tau hyperphosphorylation, which forms neurofibrillary tangles and causes neurotoxicity, leading to cognitive dysfunction.^1^ Ab plaques activate glycogen synthase kinase 3 beta (GSK3b) via the phosphoinositide-3-kinase/protein kinase B/ glycogen synthase kinase 3b (PI3K/AKT/GSK3b) signaling pathways, which leads to tau phosphorylation in neurons.^20^^,^ 22

Recently, in an effort to modify AD’s process, major advances have been made targeting amyloid-beta protein and tau-based pharmacological therapeutics. Also, deregulation of GSK-3 activity in neurons has been postulated as a key feature in Alzheimer’s disease pathogenesis. Therefore, inhibiting abnormal levels of GSK3b is a promising treatment strategy.^20^^,^ 22 Use of the MRgFUS plus MB approach holds similar promise. Many preclinical studies with animals have been conducted in a bid to establish the safety and efficacy of this method.

One of the diagnostic and therapeutic strategies for AD focuses on Ab plaque detection and reduction through active immunization (vaccination) or passive immunization (administering anti-Ab antibodies).^8^^,^ 16 FUS-MB enhances both small molecules and antibody delivery in transgenic AD mice and can be achieved even without MRI guidance in a benchtop setup. FUS-MB associated with passive immunization allows local transport of anti-Ab antibodies at high concentrations in transgenic AD mice, which could be one of the uses of this technology.^8^ When administered peripherally, the substances tend to remain in the bloodstream and only 0.1% reaches the brain;^10^ with this therapy, the percentage could rise substantially.

Relevant studies^10^^,^ 12 have shown that when FUS is used, there is an increase in BBB permeability, allowing endogenous antibodies (mainly IgG) to pass and act on the plaques, decreasing cortical Ab plaques in transgenic TgCRND8 mice. One of the studies showed that the use of MRgFUS alone reduced mean plaque size (20%) and total AB surface area (13%) within 4 days. The mechanism by which this occurs could be by the entry of endogenous antibodies, as previously explained, and by glial activation. The use of FUS alone is capable of activating glial cells without a change in their number, contributing to the clearance mechanism of Ab plaques.^12^

Although these results are interesting, complementing MRgFUS with intravenous administration of anti-AB antibodies may be required to achieve therapeutically relevant treatment efficacy. This scenario has also been tested, combining MRgFUS and exogenous antibodies.^10^^,^ 16 The passage of endogenous antibodies led to a 15% decrease in Ab plaques; however, the application of exogenous antibodies (BAM-10)^10^ led to a 61% decrease. This could be explained by the fact that this combination allows both exogenous and endogenous antibody actions.

Many of the preclinical studies demonstrated an improvement in cognition and performance of the AD animals, as illustrated throughout this review.

MRgFUS applied weekly to the hippocampus of TgCRND8^13^ mice, led to improvements in cognition, potentially mediated by reduced plaque load and increased neuronal plasticity in the dentate gyrus. The BBB was opened repeatedly in the bilateral hippocampus, a structure severely affected in AD and appropriate for clinical treatment targeting. A decrease in pathologic plaque abnormalities in the hippocampus was observed. Also, an improvement in spatial memory was shown, related to increased neurogenesis, with proliferation and maturation of newborn cells in the hippocampus, although the underlying mechanisms are unknown.

As previously discussed, tau phosphorylation is a key component in the pathophysiology of AD. In multiple preclinical models, immunization with anti-tau antibodies was shown to be effective. A group of researchers^15^ administered anti-tau scFv (single-chain variable fragment) RN2N, specific for the 2N isoform of the protein, to transgenic pR5 mice, which have overexpression of tau protein. This method showed reduction of anxiety behavior and hyperphosphorylation of tau proteins.

Given the early stage of studies, there are many variables that should be taken into consideration when evaluating the results. Some of the critical aspects that should be observed are: the targeted area, volume of the BBB opening and BBB closure time.

The fast restoration of BBB after treatment is very important and may occur in less than 12 hours,^29^^,^ 30 ^,^ 31 while the effective time for closure depends on the size of the agent being transported and also on the parameters selected to avoid damage. However, one group reported a substantially longer time to close the BBB after FUS, of about 3-5 days.^32^^,^ 33 In these studies, the larger opening was generated by higher pressures or larger microbubble diameters, probably via the larger contact area provided between the bubble and the vessel wall.^11^

O’Reilly et al.^17^ hypothesize that if the opening volume is modulated with multiple overlapping foci, the therapeutic regimen is more effective and decreases exposure, since the time for closure will become independent of the opening volume. This group of researchers also show that no differences in treatment effects were observable by magnetic resonance imaging follow-up between larger and smaller-volume sonications, suggesting that larger-volume BBB openings can be performed safely. Given that many clinical treatments would require large-volume opening of the BBB, this factor is an important consideration for clinical translation.

Another variable that may interfere in the extent of BBB opening and closing time is the brain region targeted,^9^ since it was shown that variation occurs in different regions within the same sonicated location.

Several studies have sought to optimize the parameters for BBB disruption without tissue damage. Regarding the different frequencies of FUS used, there was a preponderance of values between 1 and 2 MHz, except in a study conducted in 2010, which used a frequency of 0.588 MHz. The amount of pressure required for BBB disruption needs to be adjusted for each experiment design, since it is greater for higher frequencies.^23^ The most commonly used sonication time is 120 seconds, and the dose of injected microbubbles varies between 0.02 ml/kg and 0.04 ml/kg.^9^^,^ 12 ^-^ 14

All of the studies included in this review were in the preclinical phase and used animal models. However, trials are already underway with humans. The use of FUS-MB in humans is still in the initial phase. Only one study^34^ was found, which used 5 patients in mild-to-moderate stages of AD, with a mean of 66.2 years. The safety and efficacy related to BBB opening were the targets of the study. In all patients, there were no medium or severe consequences, and the BBB was completely closed within 24 hours. Despite the promising results concerning the study objective, the sample used was small and 4 patients were younger than 65 years old, suggesting early onset. Consequently, these two limitations restricted the generalization of the results to a larger population. Despite these limitations, the study paves the way for other more reliable studies involving larger groups.

In conclusion, the use of focused ultrasound associated with microbubbles for opening the blood-brain barrier and subsequent administration of drugs or antibodies have shown promise as a possible treatment for AD. The results of preclinical trials are positive for reduction of amyloid plaques and tau protein phosphorylation, as well as for promoting improvements in cognitive performance of AD animals.

However, a greater number of studies are necessary to systematize all the techniques used and their possible effects. Despite the promising results, the technique involves particularities and variables that require maturation for application in humans. Furthermore, the most recent study that applied the method in humans involved only a small sample with non-ideal age range. Consequently, the study failed to yield a satisfactory analysis of the results concerning safety, efficacy and benefits. This technique is new and should be further studied, as it provides a fresh perspective on the treatment of AD, a disease that causes much suffering for the many people affected and their relatives. This new understanding of a possible treatment for AD is very relevant and should be explored.
